# Gene Tagging Strategies To Assess Protein Expression, Localization, and Function in *Drosophila*

**DOI:** 10.1534/genetics.117.199968

**Published:** 2017-09-29

**Authors:** Oguz Kanca, Hugo J. Bellen, Frank Schnorrer

**Affiliations:** * Department of Molecular and Human Genetics, Baylor College of Medicine, Houston, Texas 77030; † Jan and Dan Duncan Neurological Research Institute, Texas Children’s Hospital, Houston, Texas 77030; ‡ Program in Developmental Biology, Baylor College of Medicine, Houston, Texas 77030; § Department of Neuroscience, Baylor College of Medicine, Houston, Texas 77030; ** Howard Hughes Medical Institute, Houston, Texas 77030; †† Developmental Biology Institute of Marseille (IBDM), UMR 7288, CNRS, Aix-Marseille Université, 13288, France

**Keywords:** *Drosophila*, gene tagging, genome engineering, techniques and resources, transgenesis, Flybook

## Abstract

Analysis of gene function in complex organisms relies extensively on tools to detect the cellular and subcellular localization of gene products, especially proteins. Typically, immunostaining with antibodies provides these data. However, due to cost, time, and labor limitations, generating specific antibodies against all proteins of a complex organism is not feasible. Furthermore, antibodies do not enable live imaging studies of protein dynamics. Hence, tagging genes with standardized immunoepitopes or fluorescent tags that permit live imaging has become popular. Importantly, tagging genes present in large genomic clones or at their endogenous locus often reports proper expression, subcellular localization, and dynamics of the encoded protein. Moreover, these tagging approaches allow the generation of elegant protein removal strategies, standardization of visualization protocols, and permit protein interaction studies using mass spectrometry. Here, we summarize available genomic resources and techniques to tag genes and discuss relevant applications that are rarely, if at all, possible with antibodies.

*DROSOPHILA* research has been instrumental for the functional annotation of genes and signaling pathways (Wieschaus and Nüsslein-Volhard 2016), which remains one of the most challenging endeavors of the postgenomic era. A key component of functional studies is the visualization of gene expression patterns and dynamics, and determination of the subcellular localization of the encoded proteins. In addition, live imaging methods enable the examination of protein localizations in a dynamic fashion throughout the life cycle of an organism (Sarov *et al.* 2016). Another important feature in the study of gene function relates to the identification of binding partners of a protein of interest. Indeed, biochemical studies that identify interaction partners through affinity enrichments and mass spectroscopy provide a context for the complexes in which the protein functions (Gingras *et al.* 2007). Finally, tissue-specific conditional removal of a protein followed by phenotypic analysis is critical for assessing a protein’s function (Dietzl *et al.* 2007; Taxis *et al.* 2009; Schnorrer *et al.* 2010; Caussinus *et al.* 2012; Neumüller *et al.* 2012; Chung *et al.* 2015; Nagarkar-Jaiswal *et al.* 2015b; Trost *et al.* 2016; Bence *et al.* 2017). Together, these approaches form the basis to define the molecular function of a protein of choice in an organism.

Messenger RNAs (mRNAs) as gene products are relatively simple to detect since probes for mRNA detection can be easily synthesized (Rubin *et al.* 2000; Stapleton *et al.* 2002). Hence, large-scale mRNA localization studies in *Drosophila* embryos and ovaries were conducted to document the cell and tissue types in which specific genes are expressed (Lécuyer *et al.* 2007; Tomancak *et al.* 2007; Jambor *et al.* 2015). However, these data tell us only in which cells mRNAs are expressed and provide no information about protein localization, stability, or function. Protein detection is more difficult to scale up. Previously, the detection of each endogenous protein required the generation of specific antibodies. An antibody that recognizes the endogenous protein is an invaluable tool. It can be used to report the protein expression, localization, and interactions in any genetic background. Unfortunately generating a new, specific antibody is labor intensive and does not always result in a good-quality antibody that can be used for a variety of applications. Indeed, currently < 5% of all proteins can be detected by readily available antibodies (Nagarkar-Jaiswal *et al.* 2015b). Therefore, a large-scale analysis of protein localization, stability, and dynamics is hampered by the lack of available antibodies against the vast majority of *Drosophila* proteins.

To bridge this important gap, comprehensive efforts are underway to tag proteins with well-characterized epitopes or fluorescent proteins *in vivo*, thereby preserving their endogenous expression patterns (Morin *et al.* 2001; Kelso *et al.* 2004; Buszczak *et al.* 2007; Quiñones-Coello *et al.* 2007; Venken *et al.* 2008, 2009, 2011; Lowe *et al.* 2014; Nagarkar-Jaiswal *et al.* 2015a,b; Sarov *et al.* 2016). Here, we focus on numerous tagging strategies and their advantages and disadvantages. We describe available libraries containing thousands of tagged genes and discuss applications based on these tagged genes and their encoded proteins. The long-term aim of these approaches is to generate a functional tagged protein for every fly gene and to perform *in vivo* functional characterizations of all fly proteins.

## Gene Tagging via Transgenes

Gene tagging can be accomplished either by adding the tag to the native genomic locus of the gene or by introducing a tagged transgenic copy of the gene at a secondary site in the genome. Both of those approaches depend on the ability to introduce foreign DNA into the fly genome. Here, we first introduce the basic concepts of fly transgenesis and describe different vectors and technologies used to introduce exogenous DNA into flies. This is followed by different methods concerned with how to tag genes and proteins using such transgenes. Finally, we report the recent progress toward genome-wide coverage of gene tagging using large genomic transgenes.

### Methods of transgenesis

#### Classical transgenesis methods:

The first requirement to tag a gene is the ability to introduce exogenous DNA into the fly genome. In the early days of molecular biology, only bacteria and yeast could be transformed with foreign DNA through the use of plasmids. Plasmids by their nature are exogenous, circular DNAs that bacteria and yeast can replicate and segregate during cell division. In *Drosophila*, plasmid injection was not effective as plasmids do not efficiently integrate into the genome and do not replicate extrachromosomally (Spradling and Rubin 1982).

The inability to replicate plasmids and integrate them efficiently in flies was overcome through the use of DNA transposons [also known as transposable elements (TEs)]. TEs are DNA sequences that are able to excise themselves from their genomic integration site and reintegrate at a different site, and both processes are executed by the transposase encoded within the TE (Shapiro 1983; Levin and Moran 2011). The transposase acts on transposon ends to mobilize and reinsert the transposon. To facilitate custom sequence modifications through molecular cloning and amplification in bacteria, transposon ends were integrated in plasmid backbones.

**Figure 1:**
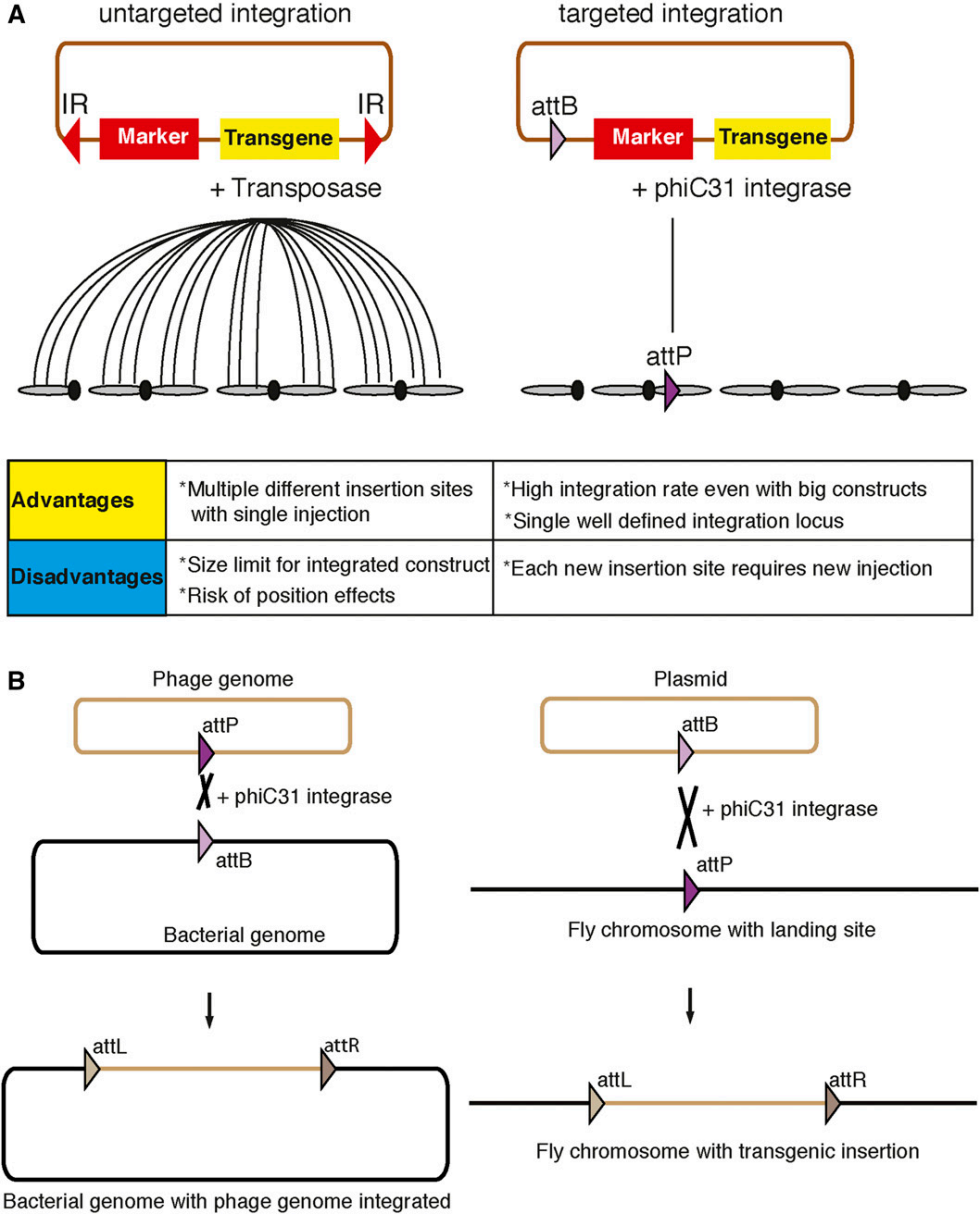
Overview of transgenesis strategies. (A) Comparison of nontargeted insertion to targeted insertion. During *P*-element transformation, the transposase integrates the plasmid containing transposon ends into the genome in an untargeted way. Multiple insertion sites can be obtained with the same injection. In φC31-mediated transgenesis, the plasmid is inserted into a specific target locus in the genome. IR, inverted repeats. (B) In the natural use of integrase, a phage genome-containing *attP* site is integrated into the *attB* site in the bacterial genome. This creates *attR* and *attL* sites that can no longer be used by the φC31-integrase enzyme as a substrate. In fly transformation, integrase enzyme integrates the plasmid-containing *attB* site in an *attP* site previously inserted in the fly genome by nontargeted insertion strategies.

By co-injection of a transposase source (a plasmid or purified mRNA encoding the transposase) and a plasmid containing a selectable marker between the TE ends into developing *Drosophila* embryos, Spradling and Rubin (1982) were able to integrate foreign sequences into the genomic DNA of some germ cells of injected embryos (Figure 1A). The ability to introduce a single copy of DNA into the fly via transposons was a paradigm shift in *Drosophila* genetics, largely due to two features of TEs. First, TEs enabled the integration of almost any DNA sequence smaller than 20 kb into the fly genome, opening the door to numerous new strategies to manipulate and test gene function in the context of the fly genome. *P*-elements were the first TE to be used in *Drosophila* for transgenesis and they remained the mainstay in *Drosophila* genetics until recently (Spradling and Rubin 1982; Bellen *et al.* 2011). More recently, piggyBac and Minos TEs have allowed the development of new tools discussed in this chapter. Second, TEs can be hopped around in the genome to induce mutations (see *Forward genetic tools*). Transposon mutagenesis facilitated the generation of new mutations and, in most cases, allowed identification of the mutated gene. Although none of the TEs insert randomly, different TEs have different biases and hence permit a broad coverage of the genome (Spradling and Rubin 1982; Bellen *et al.* 2004, 2011; Metaxakis *et al.* 2005; Venken and Bellen 2007).

#### φC31-mediated transgenesis:

TEs can integrate at different positions in the genome and the integrated DNA is therefore subject to different genetic environments. This is advantageous for generating mutations; however, it may lead to different levels of expression of the integrated exogenous sequences due to the presence of nearby enhancers or repressors. These position effects phenomena may thus compromise the comparability of different transgenes (Wilson *et al.* 1990). To minimize these local effects, the insertion should ideally be at a defined locus that is not subject to strong position effects.

Therefore, a strategy was developed to insert transgenes at specific sites in the fly genome (Groth *et al.* 2004). The technology is based on the φC31 integrase system, which inserts a plasmid containing an *attB* site into the genome at an *attP* site (Figure 1, A and B). The φC31 bacteriophage inserts its genome into an *attB* DNA sequence (*attB* site) in its host bacterial genome using the φC31 integrase and its cognate *attP* sequences in the phage genome. *attP* sites (“landing sites”) were integrated in the fly genome at numerous positions using various TEs. Hence, many transgenic fly lines containing well-characterized *attP* landing sites are available for all fly chromosomes (Groth *et al.* 2004; Venken *et al.* 2006; Bischof *et al.* 2007). The main advantages of site-specific integration are: (1) high integration efficiencies; (2) the ability to integrate different transgenes into the same *attP* landing site allowing comparable expression levels and thus reducing position effects; and (3) the integration of larger constructs with an upper limit > 100 kb (Venken *et al.* 2010).

### Transgenic tagging strategies

#### Tags:

**Figure 2:**
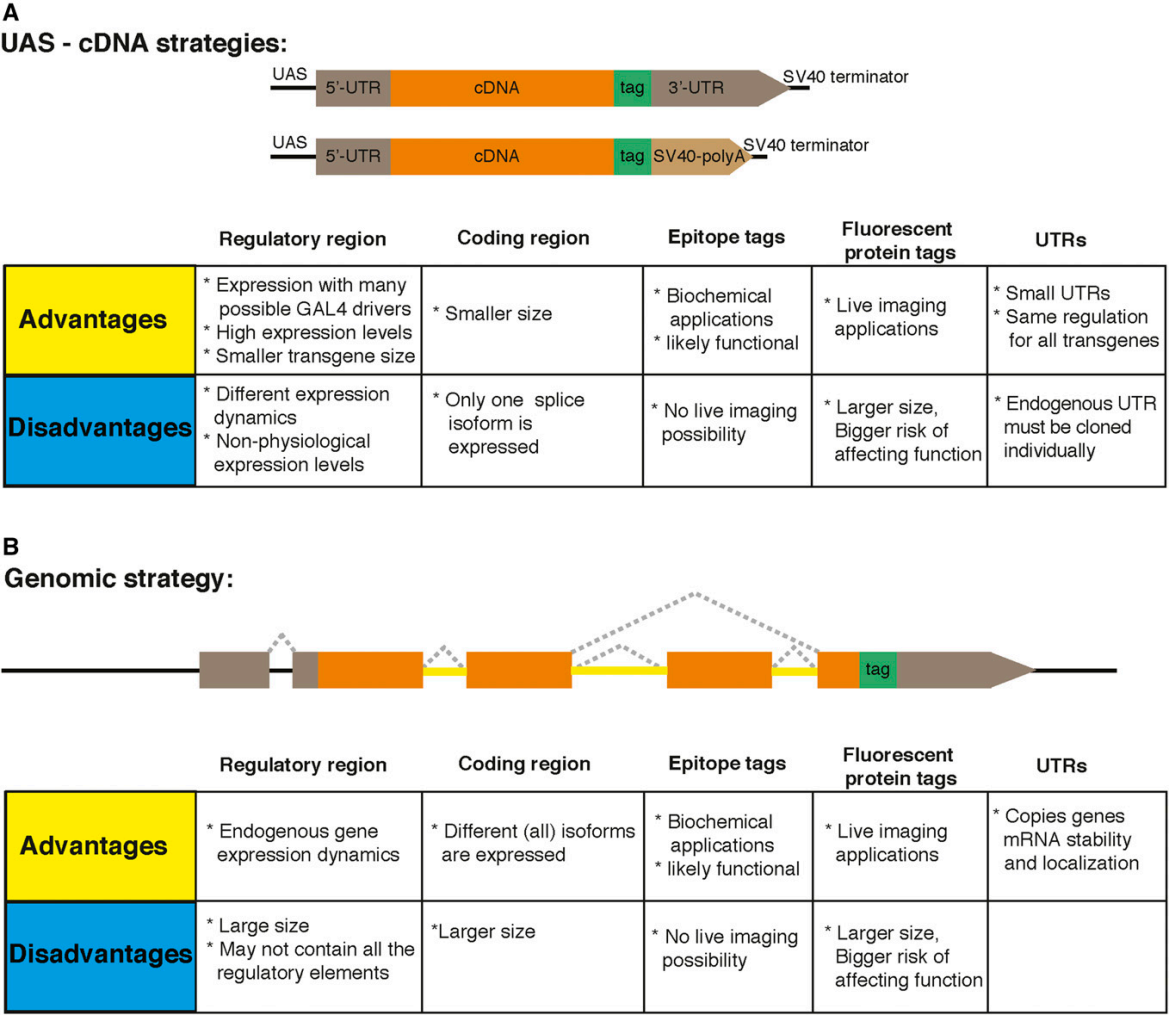
Schematics of transgenic constructs with different options. (A) Upstream activating sequence (UAS)-cDNA strategy. (B) Genomic strategy. Advantages and disadvantages of possible choices are explained below the corresponding parts of the construct. Dotted lines indicate splicing, yellow lines indicate coding introns, and black lines indicate noncoding regions. SV40, Simian virus 40.

There are multiple possible tags that can be used to tag a protein of interest, with different advantages and disadvantages depending on the application. The classical applications of tags are immunochemical detections, biochemical manipulations, and live imaging. Small epitope tags, such as Myc, Flag, V5, and HA, consist of ∼10 amino acids (aa) and are generally used for protein detection and some biochemical applications. Genetically-encoded fluorescent proteins like GFP, RFP, and mCherry are our first choice for detecting protein localization and live imaging (Figure 2). We will expand more on the applications of tags in *Applications for Epitope Tags*.

#### Classical tagging:

To tag a gene with an epitope tag followed by transgenesis, there are key elements that need to be incorporated in the transgene. These are gene regulatory regions such as enhancer/promoter that ensure transcription, 3′- and 5′-untranslated regions (UTRs) that regulate mRNA localization and stability, gene coding regions, and tag-encoding sequences (Figure 2). In general, one can use two basic strategies to tag a gene with a transgene: the first one is based on using a cDNA and the second one is based on the genomic locus containing the gene of interest. For the cDNA strategy, the cDNA is typically cloned into a transgenesis vector that contains the following DNA sequences: Upstream Activator Sequences (UAS) constituting binding sites for the yeast GAL4 transcription factor; a *Drosophila* basic promoter; a set of restriction sites allowing insertion of the cDNA; and a 3′-UTR of a gene, followed by a polyA tail (Brand and Perrimon 1993) (Figure 2A). The tag can either be incorporated after the start codon or before the STOP codon of the cDNA, generating an N-terminal or C-terminal fusion protein, respectively.

The choice of the tagging position in the protein is often tested empirically, since both N-terminal and C-terminal tags can potentially alter protein localization and/or protein function. Internal tagging of genes is also possible and is less likely to disrupt protein function (see *RMCE resources for MiMIC or CRIMIC conversions*). However, internal tags are rarely used for tagging genes in transgenic constructs, since generation of such constructs is only possible using more complicated cloning strategies. Basler and colleagues generated a collection of fly strains containing UAS-cDNA constructs for 3400 fly genes, and 2800 of those genes are tagged at the C-terminus with a triple-HA tag, enabling the systematic expression of the tagged proteins (Bischof *et al.* 2014; www.flyorf.ch).

The GAL4/UAS binary system is the most commonly used tool to express tagged proteins from cDNAs (Brand and Perrimon 1993). GAL4 is not endogenously expressed in flies and UAS sites are not endogenously present in the fly genome. Hence, GAL4 has little toxic effects and typically does not influence the expression of other genes, even if the GAL4 is expressed at high levels.

The strength of the UAS/GAL4 system is that it can be combined with a multitude of GAL4 lines that have been generated over the years. Multiple transgenic strains contain GAL4 transcription factor coding sequences under the regulatory regions of ubiquitously expressed genes such as *tubulin*, *actin*, *ubiquitin*, and *daughterless* (GAL4 drivers) to enable expression of transgenes from UAS constructs in every cell. About 400 GAL4 enhancer traps (O’Kane and Gehring 1987), which were generated by mobilizing TEs that contain a basic promotor and GAL4 coding sequences, are publicly available (Brand and Perrimon 1993; Lukacsovich *et al.* 2001; Hacker *et al.* 2003). These lines use endogenous enhancers to control the expression of UAS-cDNA constructs. Additionally, extensive libraries of transgenic fly strains that contain regulatory regions from thousands of genes were cloned upstream of a GAL4. These collections enable the expression of epitope-tagged UAS-cDNA constructs in various specific tissues or cell types (Jenett *et al.* 2012; Jory *et al.* 2012; Manning *et al.* 2012; Kvon *et al.* 2014) (http://flystocks.bio.indiana.edu/Browse/gal4/gal4_main.htm for all publicly available GAL4 fly stocks).

However, there are two important caveats to the GAL4/UAS approach: (1) unless the endogenous enhancers are present in the GAL4 line there is no information about the endogenous spatiotemporal expression pattern of the gene and (2) the subcellular protein distribution pattern must be interpreted with great caution as the GAL4 technology often causes overexpression of the tagged protein. This can lead to aberrant localization of the protein and hence create confounding issues.

The amount of expressed protein and the timing of induction can be varied using the GAL4/UAS system. Reducing the temperature at which the flies are kept generally reduces the expression of transgenes as many, but not all, GAL4 binary systems show temperature dependence (Duffy 2002). Ligand-dependent variants of GAL4 can be regulated by the amount of ligand (Han *et al.* 2000; Osterwalder *et al.* 2001; Roman *et al.* 2001); however, only a few tissue-specific versions of such GAL4 variants are available. Moreover, GAL4 activity can be suppressed efficiently by the use of GAL80, which is a GAL4-specific repressor (Lee and Luo 1999). A temperature-sensitive version of GAL80, GAL80^ts^, is available that can rapidly be inactivated by a temperature shift (McGuire *et al.* 2003). The use of GAL80 or GAL80^ts^ are thus good options to attenuate GAL4/UAS-based expression levels; however, they increase the number of transgenes that need to be crossed together.

A second tagging approach is to create a tagged copy of the genomic locus of the gene of interest (Figure 2B). In general, this is the preferred strategy as the expression pattern and dynamics for such tagged mRNAs and proteins are very similar or identical to the wild-type protein encoded by the endogenous locus, based on comparisons with antibody stainings (Venken *et al.* 2006). In brief, a genomic fragment from the fly genome that contains the protein-coding sequence of a gene, together with its putative regulatory regions (genomic enhancers, promoter, and 5′- and 3′-UTRs) is cloned and a tag coding sequence is inserted into this fragment. Ideally, the DNA between the 5′ and 3′ ends of the gene of interest and the next 5′ and 3′ genes in the genome should be part of the transgene. For genes that span < 20 kb (including their UTRs) this is often feasible with *P*-elements, but traditional cloning methods and transgenic insertion of DNA using TEs becomes inefficient when the integrated DNA exceeds 20 kb.

#### Tagging by recombineering:

By design, the classical tagging approach in *Drosophila* using the GAL4/UAS system does not reflect the expression pattern of the gene and, in some cases, the subcellular localization of the protein thus provides limited information. Therefore, it is desirable to express the tagged protein under endogenous control by including as many regulatory elements as possible. This can be done by tagging the endogenous gene through homologous recombination (which will be discussed in *Tagging Endogenous Loci In Vivo*) or by using large genomic transgenes. A genomic transgene includes the genomic sequence of the gene (including 5′- and 3′-UTRs), which will most often respect splicing regulation, as well as mRNA localization and stability. Additionally, such a construct must contain the necessary 5′- and 3′-flanking sequences to include enhancers of the gene. This may require a large DNA construct of 20–30 kb or larger, which cannot be easily manipulated with classical cloning methods. The method of choice to insert a tag into a given gene within such a large construct is “recombineering” in *Escherichia coli*. After successful tagging, the large construct is transformed into flies by φC31-mediated site-directed transgenesis (Venken *et al.* 2006).

**Figure 3:**
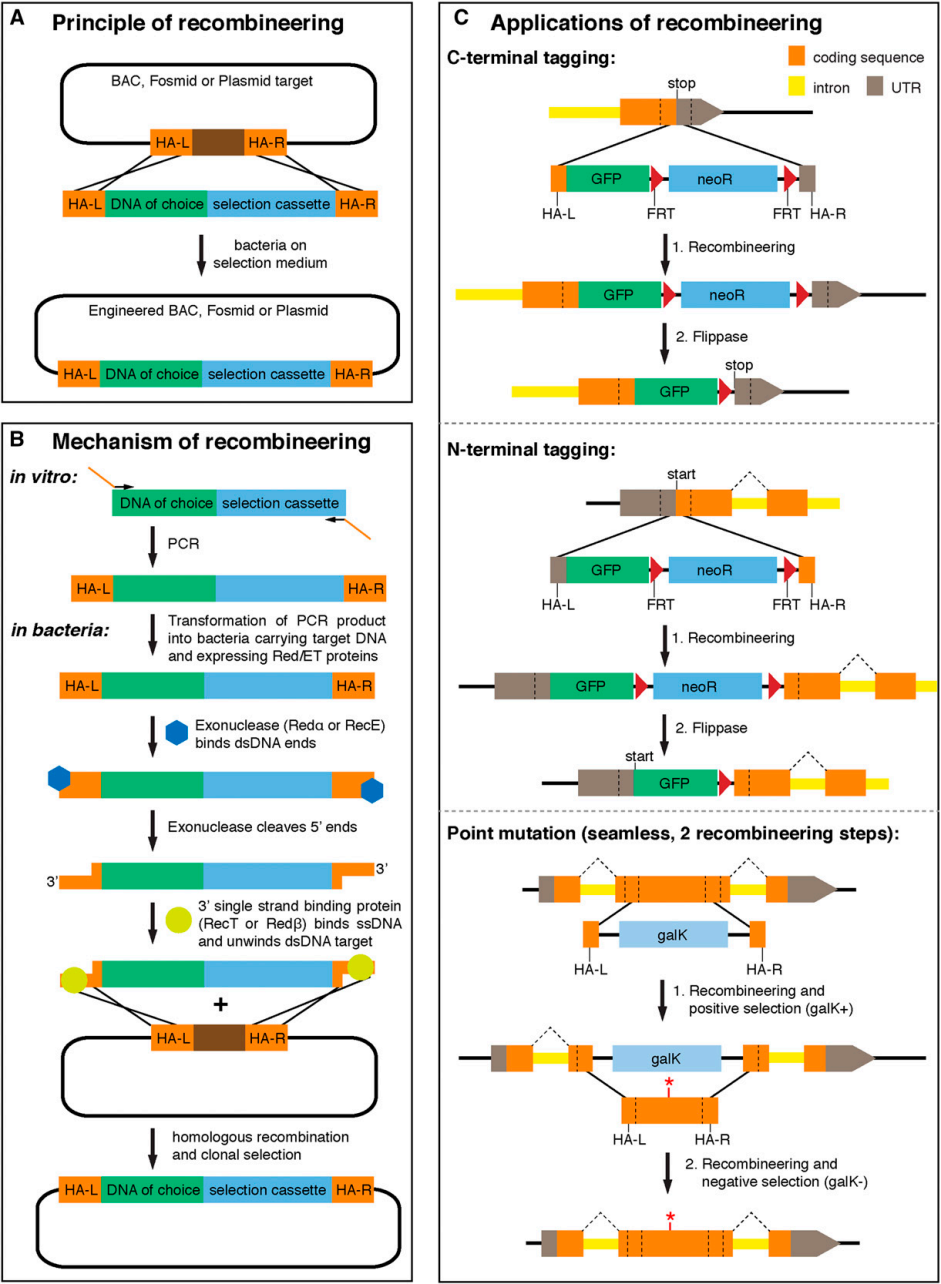
Overview of recombineering principle and its applications. (A) The basic principle of recombineering: a linear DNA piece containing homology arms (HA-L and HA-R) and a selection marker is inserted precisely into a circular target DNA. (B) Molecular mechanism of recombineering: 5′ to 3′ exonuclease activity and single-strand annealing mediated by the Red/ET system. (C) Applications of recombineering: simple scar-containing variant for C- and N-terminal tagging followed by Flp-induced removal of the selection cassette *vs.* two-step recombineering for seamless integration of a point mutation (red asterisk). (B) was modified from Sharan *et al.* (2009).

Recombineering uses *in vivo* homologous recombination in *E. coli* to insert DNA sequences into other large DNA molecules with high fidelity (Figure 3A). It was historically developed to engineer the *E. coli* genome (Murphy 1998; Zhang *et al.* 1998). However, it can also be applied to engineer other circular DNAs, plasmids, fosmids, or bacterial artificial chromosomes (BACs) in *E. coli*. Therefore, recombineering can be widely applied for gene tagging unconstrained by available restriction sites and construct size.

*E. coli* strains used for molecular biology are generally defective in homologous recombination and maintain a stable genome, including transformed circular DNA molecules. The Red/ET system was developed to induce controlled homologous recombination in *E. coli*. It utilizes the function of two proteins, either derived from the λ phage [λ-Redα and Redβ proteins (Murphy 1998)] or the Rac prophage [RecE and RecT (Zhang *et al.* 1998)]. Prior to the recombineering event, expression of both proteins is induced in bacteria carrying the target DNA. Then, a linear double-stranded DNA molecule with homology to the target sequence is transformed into *E. coli* to modify the target construct. The double-stranded DNA will be bound by the first protein, a 5′ to 3′-exonuclease (Redα or RecE). This cleaves 5′ ends and produces 3′ overhangs at both ends. These 3′ overhangs are bound by the second protein, a single-strand-binding protein (Redβ or RecT), which also mediates the unwinding and annealing of homologous sequences in the double-strand DNA substrate present in the vector. This triggers homologous recombination resulting in the precise insertion of the linear DNA into the target DNA (Figure 3B).

The strength of this method is that both the substrate and target DNA can contain almost any DNA sequence. Recombineering only requires short, usually 50-bp long homology arms (HA-L and HA-R) on each side of the linear DNA and a selection marker within the inserted sequence to identify the positive events (Figure 3, A and B). This linear DNA can be easily produced by Polymerase Chain Reaction (PCR) *in vitro* with two 70-bp long primers, adding the specific homology arms to a common selection cassette, typically an antibiotic resistance gene that helps identify successful recombineering events. The target DNA can be a plasmid, fosmid, or even a large BAC, with the only requirement being that the target is present at low copy during the recombineering process (Muyrers *et al.* 1999; Sharan *et al.* 2009; Ejsmont *et al.* 2011a). Recombineering is efficient and precise, and very few insertions or deletions are introduced at the breakpoints. It is ideally suited to tag *Drosophila* genes present in large genomic clones (Venken *et al.* 2006).

How can recombineering be practically applied to efficiently tag fly proteins? There are two distinct variants using double-stranded DNA as a recombineering template. The simple one leaves the selection cassette or at least a small scar within the inserted DNA, whereas the more complex one is seamless but requires two recombineering steps (Figure 3C). In the simple variant, the selection cassette is usually flanked by site-specific recombinase target FRT or loxP sites and inserted together with the tag sequences by recombineering. After successful clonal selection, the expression of the recombinase, by either transforming bacteria with recombinase coding sequence-containing plasmids or inducing recombinase gene expression previously introduced into the bacterial genome, can be induced and the selection marker can be removed. In this scenario, a FRT or loxP site remains that is generally translated after the inserted tag sequence and fused with the protein of interest (Figure 3C). Such a small scar is normally tolerated, as a tag by nature also inserts a foreign sequence into the protein of interest. This simple variant is suitable for high-throughput tagging of proteins, both at the N-terminus or the C-terminus (see *The fly transgeneOme clone library* for more practical details).

The second recombineering variant results in a seamless product without any unwanted DNA sequences left in the final construct. It requires two recombineering steps; the first inserts a positive selection marker at the position of choice again using short homology arms, while the second recombineering step removes this selection marker by a counter selection procedure and instead inserts a sequence of choice such as a tagged exon or a mutated exon (Figure 3C). Selection markers that are suitable for positive selection and counter selection in bacteria include the *galK*, *rpsL*, and *mfabI* makers (Warming *et al.* 2005; Wang *et al.* 2009; Jang and Magnuson 2013). Counter selection is generally more problematic than positive selection, which makes this variant less suitable for high-throughput applications and more suitable for those that require the tag to be located at specific positions (Klose *et al.* 2013).

Finally, another approach uses a single-strand oligonucleotide as the recombineering template, without a positive selection marker. This variant can be used to insert point mutations or to create precise deletions. Since the insertion of nonhomologous DNA is limited to 10–20 bp, and the detection of insertion requires laborious PCR screening, this approach is less attractive for systematically tagging proteins (Sawitzke *et al.* 2013).

### Genomic libraries

To utilize genomic libraries for gene tagging efficiently, it is important to consider the size of the fly genes and estimate the regulatory regions required to achieve endogenous expression. The *Drosophila* genome contains ∼117 Mb of euchromatin, coding for > 14,000 protein-coding genes (Brown *et al.* 2014). The average gene contains four exons with an average gene body length (genomic region including 5′- and 3′-UTRs) of ∼3 kb. Together, all gene models occupy ∼44 Mb of the fly genome (Hoskins *et al.* 2015). When considering 10 kb upstream and 5 kb downstream of the gene model as sufficient to cover most of the regulatory regions and preserve endogenous expression levels, ∼90% of all genes can be covered with 36 kb of genomic DNA (Ejsmont *et al.* 2009). Hence, only a minority of genes should require larger clones for complete coverage.

To accommodate the different sizes of genes, several *Drosophila* genomic libraries have been developed (Table 1). They are either based on BACs, which are very flexible in size and can host genomic DNA larger than 300 kb (Shizuya *et al.* 1992), or so called fosmid vectors, which during preparation require packing of the genomic DNA into a λ phage particle resulting in an insert size range of 25–45 kb (Ejsmont *et al.* 2009). Faithful segregation of these large vectors requires an origin of replication to keep the BAC or fosmid at a single copy (often derived from the *E. coli* F-factor). However, most practical are vectors that carry two origins of replication (like *oriV* and *oriS*), which enable a controlled induction to high copy number, thereby combing faithful segregation and high-efficiency recombineering with DNA amplification for efficient DNA isolation (Wild *et al.* 2002).

**Table 1:** *Drosophila* genomic clone libraries and their applications

Library name	Vector type	Clone number (ends sequenced)	Average insert size (kb)	Gene coverage (%)	Tagged?	Vector features	Applications	Marker
CHORI-322	BAC (P[acman])	52,081 (5 × coverage)	21	88.9	Untagged	attB; high-copy inducible	Tagging of small genes	*white +*
CHORI-321	BAC (P[acman])	23,899 (9 × coverage)	83	99.3	Untagged	attB; high-copy inducible	Tagging of large genes; X chromosome duplication	*white +*
RPCI-98	BAC	17,540 (24 × coverage)	163	> 99	Untagged	Low-copy only	DNA source for gap repair recombineering	Not suited for transgenesis
FlyFos	Fosmid (FlyFos)	15,204 (3.3 × coverage)	36	89.3	Untagged	attB; high-copy inducible	High-throughput tagging	3 × P3-dsRed
Pretagged- TRG	Fosmid (FlyFos)	11,257 (not sequenced)	36	84.0	Spacer inserted for tagging	attB; high-copy inducible	High-throughput tagging	3 × P3-dsRed
sGFP-TRG	Fosmid (FlyFos)	9,580	36	71.5	GFP-tagged (C-terminal)	attB; high-copy inducible	Rescue, protein localization, purification	3 × P3-dsRed
FlyFos *D. pseudoobscura*	Fosmid (FlyFos)	2,592	36	37	Untagged	attB; high-copy inducible	*Trans*-species rescue (for RNAi rescue)	3 × P3-dsRed

The summary of all available genomic clones, their coverage, and intended applications. RNAi, RNA interference.

Generally, smaller genomic clones are easier to work with as fly transgenesis is less efficient for large clones (Venken *et al.* 2009). Hence, larger clones are only preferred for tagging complex genes or for generating larger chromosomal duplications (Venken *et al.* 2010). The very large RPCI-98 BAC clones, which were used for assembling the fly genome, do not contain an *attB* site (Hoskins *et al.* 2000). Although, these cannot be directly used for transgenesis (Table 1), they can be retrofitted by integration of an *attB* site (Kondo *et al.* 2009). This strategy has been successfully used to create a series of 200-kb clones covering large sections of the 4th chromosome available at the Bloomington *Drosophila* Stock Center (BDSC) (K. Venken, personal communication).

#### P[acman] libraries:

Theoretically, recombineering can be used to retrieve large DNA fragments from already available BAC libraries. A retrieved gene can be tagged by a second round of recombineering and integrated into the fly genome. In practice, the efficiency of retrieving large DNA fragments and recombineering are hampered by the high copy number of transposon vector backbones. Although high copy number vectors are very useful to obtain enough DNA for conventional cloning, sequencing, and injection, they are not useful for recombineering. Recombineering efficiency, as well as the maximum size that can be integrated by gap repair, drops very significantly with high copy number plasmid backbones (Copeland *et al.* 2001; Lee *et al.* 2001).

To optimize recombineering and transgenesis of large constructs in the fly genome, “P[acman]”, a BAC-type plasmid was developed (Venken *et al.* 2006). The P[acman] vector contains two origins of replication to allow copy number control and an *attB* site to integrate its content at defined *attP* sites into the fly genome for transgenesis. These two features help to boost transgenesis efficiency.

The P[acman] methodology was initially demonstrated by recombineering 17 genes from large genomic BAC clones into the P[acman] backbone by recombineering, followed by the generation of transgenic flies. Fragments up to 133 kb were recombineered and integrated into the fly genome (Venken *et al.* 2006). Multiple vectors containing diverse tags that can be amplified by PCR to tag genes at the N- or C-termini were generated to facilitate gene tagging by recombineering using the P[acman] platform (Venken *et al.* 2008). In a follow-up study, P[acman] clones were used to tag 20 genes. In 19 cases tested, tagged constructs were expressed and recapitulated the known expression patterns of endogenous genes (Venken *et al.* 2009). The only gene that failed to completely recapitulate the endogenous expression pattern had an enhancer that was too distant in the genome to be included in the initial P[acman] construct. Inclusion of this enhancer in the transgene was sufficient to recapitulate the full endogenous expression pattern. Three tagged transgenes were further studied and tested for functionality. All three rescued the phenotypes associated with loss-of-function of the respective endogenous genes (Venken *et al.* 2008, 2009). These experiments demonstrated that tagging by recombineering using the P[acman] method is feasible for most genes.

After these proof-of-principle experiments, two large P[acman]-based BAC libraries were generated. These contain genomic fragments with an average size of 21 kb for CHORI-322 or 83 kb for CHORI-321 (Table 1). Combined, > 75,000 clones were sequenced, covering > 99% of all the annotated genes (Venken *et al.* 2009). These clones can be successfully inserted into the fly genome, although transgenesis efficiency is lower for the 80-kb clones (Venken *et al.* 2010).

Large genomic clones often contain multiple genes. Hence, transgenesis with these large clones often leads to duplication of numerous genes. Theoretically, this may cause unwanted phenotypes or even lethality and limit the use of genomic clones for tagging applications. Another potential risk is that the genomic constructs inserted in different genomic contexts may not be expressed as efficiently as the endogenous counterparts. To systematically assess both caveats, 408 overlapping clones from both P[acman] libraries, which together almost span the entire sequenced portion of the X chromosome, were integrated into a third chromosome *attP* landing site in flies (Venken *et al.* 2010). This transgenic fly collection was the first molecularly-defined set of strains that duplicates an entire chromosome in a eukaryotic model organism. The functionality of this collection was tested by rescue of mutations in genes resulting in visible or lethal phenotypes. The duplication collection rescued 92% of the 158 tested mutations, illustrating the usefulness and functionality of the collection. Those experiments showed that the majority of X chromosomal genes are remarkably robust in tolerating a copy number increase associated with the BAC duplication set (Venken *et al.* 2010). Together with previous observations that *Drosophila* can tolerate large X ray-induced chromosomal duplications, this collection showed that large genomic transgenes can be used to express genes from other chromosomal positions. Moreover, recent commercial efforts have generated defined P[acman]-based duplications for ∼50% of the second and the third chromosomes. These are available through Genetivision (http://www.genetivision.com/duplication.html).

The P[acman] BAC libraries greatly facilitate the mutagenesis and tagging of genes of interest with any desired tag. Indeed, multiple P[acman] clones have been ingeniously used to conduct structure function analysis (Enneking *et al.* 2013; Siegenthaler *et al.* 2015; Stephan *et al.* 2015; Cichewicz *et al.* 2017; D’Brot *et al.* 2017) and tagging approaches (The modENCODE Consortium *et al.* 2010; Negre *et al.* 2011; Gerlach *et al.* 2014; Hayashi *et al.* 2016; Nashcheckin *et al.* 2016; Corson *et al.* 2017). P[acman] BACs containing the genes of interest are available through BACPAC resources (http://bacpacresources.org). Most BACs are currently untagged, but they can be tagged by recombineering using available plasmids, since the genomic insert contains the regulatory elements of the tagged gene and the tagged transgenes very often mimic the endogenous expression of the gene of interest.

#### Drosophila fosmid libraries:

**Figure 4:**
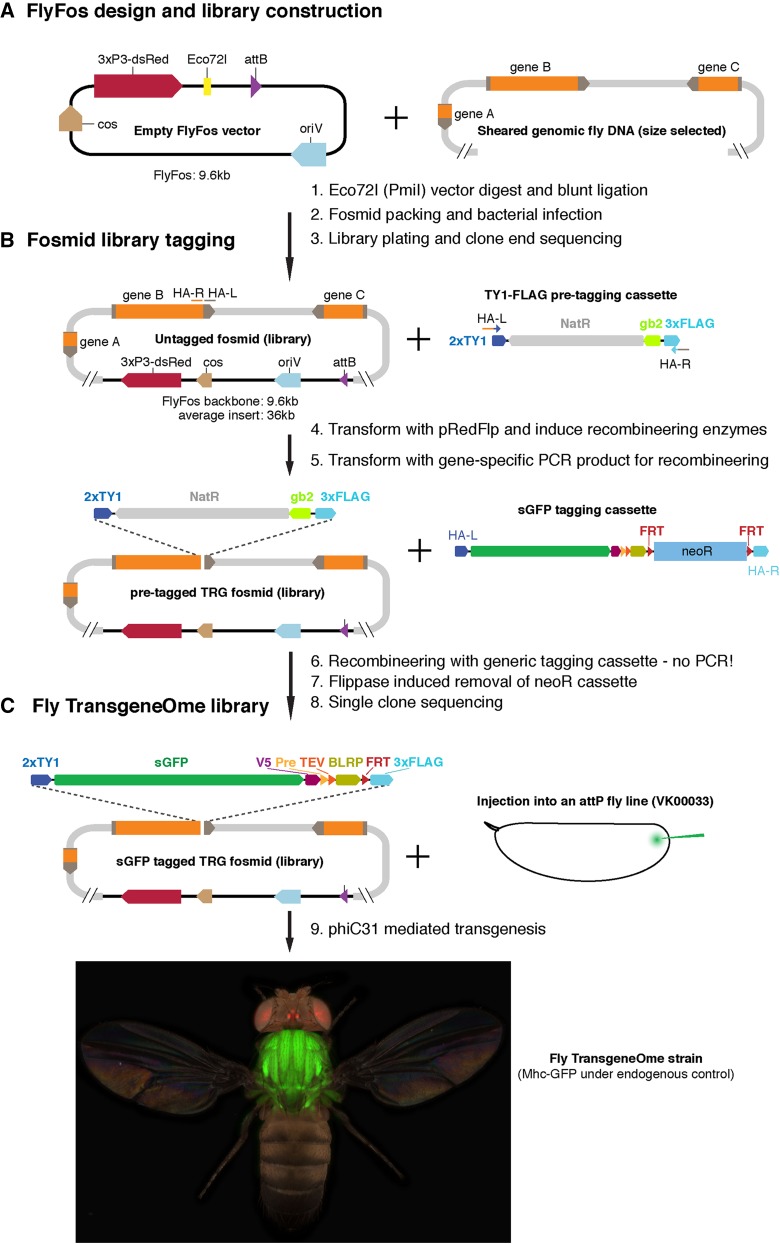
Fosmid library preparation, tagging, and transgenesis workflow. (A) FlyFos design and library construction. (B) Two-step fosmid tagging strategy, first generating the pretagged TRG fosmid clone library and second the sGFP-tagged TRG fosmid clone library. (C) Transgenesis step to build the fly TRG strain library. Note the expression of the dsRed marker in the adult eyes and the sGFP in the muscles, in particular the flight muscles, of this strain (tagged myosin heavy chain-sGFP protein, fTRG500; we acknowledge the help of Nicholas Gompel for acquiring the fly image). sGFP, superfolder GFP; TRG, TransgeneOme.

Similar to BAC-based libraries, fosmid-based libraries also house large pieces of genomic DNA. An important distinction is that fosmid libraries are generated by packing the ligated DNA (the fosmid backbone and genomic insert) into a λ bacteriophage, which effectively infects *E. coli* cells to generate large numbers of clones (Figure 4A). This restricts the size of the genomic insert to ∼45 kb (Ejsmont *et al.* 2009).

A commonly used fosmid backbone is called FlyFos that, similar to P[acman], contains the following important features (Figure 4): (1) An *oriV* origin of replication, in addition to the normal F-plasmid origin, enables the flexible switch from single copy to high copy by the simple addition of arabinose to the medium, thus fosmids are also stably segregating in bacteria, well-suited for recombineering in the single copy mode, and can be induced to high copy for efficient fosmid isolation; (2) an *attB* site enables efficient integration of the fosmid into any *attP* site in the fly genome; (3) a 3 × P3-dsRed eye marker, derived from the enhancer of the *eyeless/Pax-6* gene, a highly expressed marker that can be easily detected; and (4) a *cos* sequence for efficient viral packing. The features of FlyFos allowed the generation of *Drosophila* fosmid libraries (Ejsmont *et al.* 2011b).

The current *Drosophila melanogaster* FlyFos library consists of ∼15,000 sequenced clones, with an average insert size of 36 kb. It covers 89% of the *Drosophila* genes, if one considers 10 kb upstream and 5 kb downstream of the gene as the typical expanse of a gene (Ejsmont *et al.* 2009) (Table 1). Proof-of-principle experiments using a liquid recombineering strategy for gene tagging (see *The fly transgeneOme clone library*) showed that eight out of twelve tagged fosmid clones recapitulate the endogenous expression pattern of the gene, showing that the FlyFos library is a useful tool for functional studies (Ejsmont *et al.* 2009).

### The fly transgeneOme (TRG) clone library

As the FlyFos library was shown to contain functional copies of the fly genes and recombineering strategies improved (Sarov *et al.* 2012), it became feasible to design an optimized recombineering strategy using liquid culture in 96-well plates for systematic genome-wide tagging. This strategy used a “pretagging” strategy, in which a simple selection marker is inserted as a “pretag,” which can be very effectively replaced by tags of choice in a second recombineering step (Hofemeister *et al.* 2011). The key steps are as follows. (1) To improve recombineering efficiency, a vector (pRedFlp, which confers hygromycin resistance) containing both the Red recombinase components and Flippase to remove the selection marker (see Figure 3) was constructed. In this vector, the Red enzymes are under tight expression control (regulated by the addition of rhamnose) to ensure only a brief pulse of recombinase expression during the recombineering step. This is important to maintain genome and plasmid integrity. (2) The pRedFlp was transformed into the fosmid in 96-well plates and bacteria were selected by the addition of hygromycin in liquid culture. (3) A pretag containing a 2 × TY1 tag, a selection marker, and a 3 × FLAG tag was constructed. The pretag was amplified by PCR with gene-specific primers, containing 50-bp gene-specific overhangs for C-terminal tagging, and transformed into fosmid- and pRedFlp-containing bacteria, in which the recombinase enzymes had been induced. Antibiotic selection resulted in the growth of the positive clones with the C-terminal pretag (Figure 4B). More than 11,000 pretagged clones were generated in liquid culture: the pretagged TRG clones (Table 1). The recombineering events in these cultures were not sequence verified individually and the clones were used to proceed to the second tag exchange step directly (Sarov *et al.* 2016). For the tag exchange, a multi-functional 2 × TY1, superfolder GFP (sGFP), V5, precision and TEV (Tobacco Etch Virus) protease cleavage sites, BLRP (Biotin Ligase Recognition Peptide), 3 × FLAG tag containing a neomycin selection marker was excised from a plasmid and used as recombineering template. The short 5′ and 3′ homology arms consisting of the 2 × TY1 and 3 × FLAG tag are sufficient for efficient recombineering. Clones were selected for neomycin resistance in liquid culture and the neomycin cassette was excised by induction of Flippase. The pRedFlp cassette is then lost by a shift to high temperature, avoiding chromosomal instabilities due to unwanted recombinase expression. Finally, the cultures were plated and single clones were sequenced. More than 9500 verified sGFP-tagged fosmid clones were isolated constituting the first systematic genome-wide tagged *Drosophila* library, the sGFP-TRG clone library (Sarov *et al.* 2016) (Figure 4B and Table 1). The existence of a pretagged library enables a fairly simple exchange to other tags of choice, such as a self-cleaving 2A peptide fused to a superfolder GFP with a nuclear localization sequence (T2A-sGFPnls) T2A-sGFPnls transcriptional reporter tag, which was tested at a small scale (Sarov *et al.* 2016).

The fosmid tagging strategy works very reliably to tag most genes covered by the library, currently comprising 11,745 genes, of which ∼9500 were successfully tagged. C-terminal tagging was chosen as only 1400 fly proteins contain alternative C-termini, resulting in tagging of all protein isoforms for 90% of the fly genes (Sarov *et al.* 2016). As the tag is inserted in the coding sequence prior to the endogenous STOP codon, ∼3000 genes that do not contain an intron flanked by two coding exons (coding intron) can also be tagged (Sarov *et al.* 2016), a feature that is not easily achieved with other methods [see *Minos-Mediated Integration Cassette (MiMIC)*]. In summary, the pretagged and sGFP-tagged TRG fosmid libraries constitute a highly versatile resource for the fly community. The clones are commercially available at Source Biosciences (https://www.sourcebioscience.com/products/life-science-research/clones/transgenome-resources/drosophila-transgenome-resource/).

### The fly TRG strain library

**Figure 5:**
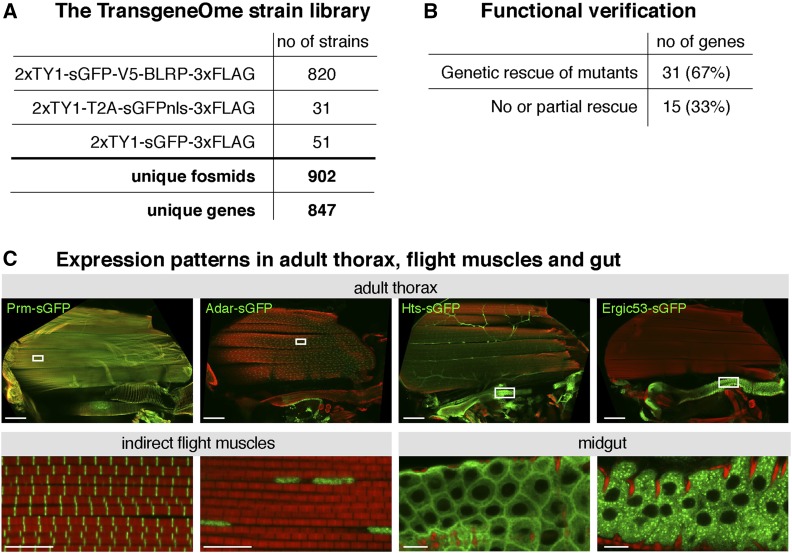
Overview of fly TransgeneOme strain library. (A) Number of available fly strains with the respective tags. (B) Functional verification by genetic rescue. (C) Collection of expression patterns in the adult fly thorax for Prm-sGFP (fTRG475, localizes at sarcomeric M-line in flight muscles), Adar-sGFP (fTRG570, localizes in nuclei of all cells), Hts-sGFP (fTRG585, expressed in motor neurons and present at the cortex of the gut epithelium) and Ergic53 (fTRG158, localizes in vesicles in the gut epithelium). GFP is shown in green and actin with phalloidin in red. Size bars are 100 µm in thorax and 10 µm in the enlarged boxes. We acknowledge the help of Christiane Barz for acquiring the images.

The sGFP-fTRG fosmid clone library was used to generate a transgenic genomic tagged library, the fly TRG strain library. In total, transgenic lines for 847 different genes were generated and deposited at the Vienna *Drosophila* Resource Center stock center (http://stockcenter.vdrc.at/) (Sarov *et al.* 2016). This collection is regularly updated. Most lines were generated by inserting the tagged fosmid into the landing site VK00033 at position 65B on the third chromosome (Venken *et al.* 2006). The majority of lines (820) were generated with the 2 × TY1-sGFP-V5-Pre-TEV-BLRP-3 × FLAG tag (Figure 5A). This tag enables both live imaging due to a particularly fast maturing GFP (sGFP) (Pédelacq *et al.* 2006) and biochemical purification with any of the small epitope tags or the GFP. It also allows multistep purifications using the precision or TEV-protease cleavage sites (Rigaut *et al.* 1999; Puig *et al.* 2001) or selective biotin labeling with the BLRP peptide (Deal and Henikoff 2010).

To assess the functionality of the fTRG-tagged fosmids, a selection of 46 tagged genes, for which well-characterized classical mutants existed, were chosen and the tagged fTRG transgenes were crossed into the mutant background. Two-thirds of the transgenes rescued the corresponding mutants (Sarov *et al.* 2016) (Figure 5B). Most of the tested genes, which failed to rescue, were transcription factors (10 of 15) with multiple roles during development. They often contain a complex enhancer landscape that may not be included in the 36-kb genomic DNA present in the fosmid. The multifunctional tag of the sGFP-TRG library enables multiple applications, including biochemical complex enrichments followed by mass spectrometry and protein visualizations in live or fixed tissues (see *Applications for Epitope Tags*). Hence, this resource is a highly valuable tool for the functional characterization of fly genes.

An intrinsic limitation of the fosmid library is its insert size limit, which excludes the functional tagging of large genes with large regulatory elements. These can be tagged by recombineering using the larger P[acman] BACs or by modification of the endogenous locus (see *Tagging Endogenous Loci In Vivo*).

## Tagging Endogenous Loci In Vivo

So far, we have focused on methods to integrate tagged versions of a gene of interest at secondary sites in the genome using transgenesis. A more elegant alternative is to tag the gene of interest at its endogenous location. Tagging the gene of interest at its genomic location offers both forward genetic and reverse genetic tools to manipulate the locus of interest.

### Forward genetic tools

#### Protein trapping with P-elements and PiggyBacs:

Protein trapping is an untargeted insertion strategy that aims to insert a tag sequence into protein-coding regions of genes. In *Drosophila*, this is accomplished by nesting an artificial exon in a transposon backbone (Morin *et al.* 2001; Clyne *et al.* 2003; Kelso *et al.* 2004; Buszczak *et al.* 2007; Quiñones-Coello *et al.* 2007; Lowe *et al.* 2014; Nagarkar-Jaiswal *et al.* 2015b). Typically, the artificial exon contains a GFP lacking start and stop codons that is flanked by a set of powerful splice acceptor (SA) and donor (SD) sites. When this transposon is inserted in an intron between two exons in the SA-GFP-SD orientation, the artificial exon is spliced into the mature mRNA of the gene. If the artificial exon is inserted in an intron separating two coding exons (coding intron) in the proper orientation, it will be translated. For the translation to result in a functional tag, the artificial exon should follow the open reading frame corresponding to the reading frame of the preceding exon. Hence, only one of six insertions in coding introns functions as a protein trap.

Initial screens used *P*-element vectors for protein trapping. To account for each possible reading frame, three different transposons were mobilized in each screen. *P*-elements have a very strong insertion bias for promoters rather than in introns (Spradling *et al.* 1995, 2011; Bellen *et al.* 2004, 2011). Hence, the use of PiggyBacs that were shown to have less insertional specificity was explored. Unfortunately, PiggyBacs are more difficult to mobilize than *P*-elements. These constraints limit the number of useful insertions to 1 in 2000 animals for *P*-elements and 1 in 50,000 when PiggyBacs are used (Kelso *et al.* 2004; Buszczak *et al.* 2007). This low frequency of useful inserts severely limits the number of genes that can be tagged by these transposons. Indeed, analysis of the Kelso *et al.* (2004) and Buszczak *et al.* (2007) data showed that only 226 unique genes contain functional protein traps (Aleksic *et al.* 2009). A more recent screen, using a PiggyBac/*P*-element hybrid element, screened 41 million animals and tagged and documented the expression and subcellular localization of an additional 250 genes (Lowe *et al.* 2014). In summary, these strategies are not efficient and only 500–600 different genes have been tagged using this methodology (http://cooley.medicine.yale.edu/flytrap/).

#### Minos-mediated integration cassette (MiMIC):

**Figure 6:**
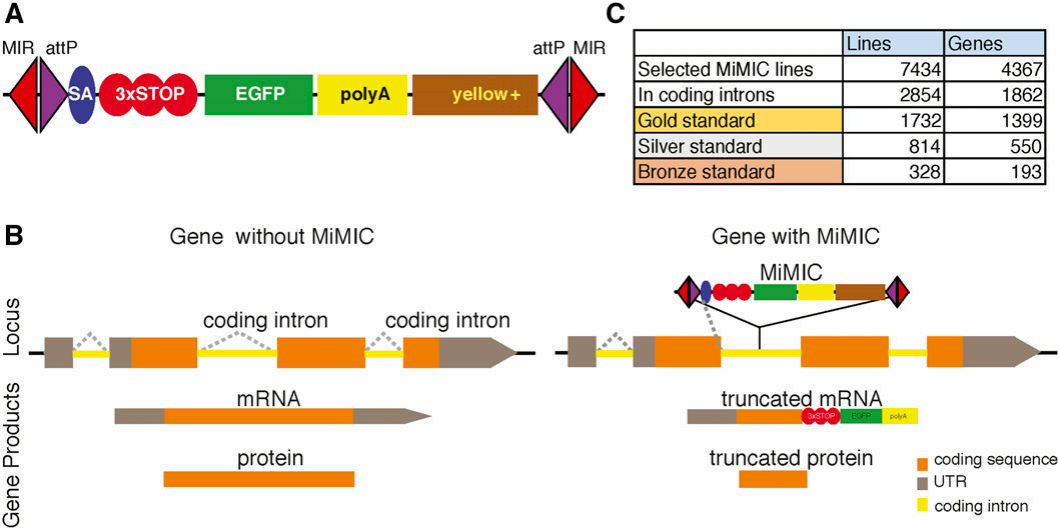
Overview of MiMIC (Minos-Mediated Integration Cassette) lines. (A) Schematics of MiMIC construct. MIR, Minos Inverted Repeats; SA, Splice Acceptor; 3 × STOP, stop codons in all possible open reading frames; yellow+, mini yellow dominant selection marker. (B) Schematic of genomic region with or without MiMIC. Dotted lines indicate splicing. (C) MiMIC collection numbers.

Minos is a relative newcomer to the *Drosophila* transposon repertoire when compared to *P*-elements and PiggyBacs (Metaxakis *et al.* 2005). Analysis of the distribution of Minos insertions showed that they are much better suited for protein trap approaches, as Minos integrates almost at random in the genome and even shows a subtle preference for introns (Metaxakis *et al.* 2005; Venken *et al.* 2011). MiMIC is a Minos-derived transposon engineered to contain a mutator cassette and a dominant marker (yellow), nested between two inverted φC31 *attP* sites. The mutator cassette has a powerful splice acceptor followed by stop codons in the three reading frames and a polyA tail. The polyA tail arrests transcription of the message and truncates the mRNA at the site of the MiMIC insertion (Figure 6, A and B). Depending on the insertion site, a MiMIC insertion can affect either all the isoforms or a subset of the isoforms of a gene. MiMICs that affect all annotated isoforms were dubbed Gold, those that affect > 50% of the annotated isoforms are Silver MiMICs, and those that affect only one or few isoforms are Bronze (Figure 6C). The mutator cassette also contains a cryptic ribosomal initiation site before the Enhanced Green Fluorescent Protein (EGFP) sequence. This EGFP does not play a role in the mutagenic activity of MiMIC and typically is not expressed from MiMICs inserted in coding introns. However, the EGFP serves as a gene trap signal for MiMICs inserted in the 5′-UTR (Venken *et al.* 2011). Expression of φC31 integrase allows the exchange of sequences contained between the two *attP* sites with any DNA sequence nested between the *attB* sites (Groth *et al.* 2004), a process called Recombination-Mediated Cassette Exchange (RMCE) (see *RMCE resources for MiMIC or CRIMIC conversions*).

MiMICs are highly mutagenic when inserted in coding introns in the same transcriptional orientation as the host gene but are not mutagenic when integrated in the opposite orientation (Nagarkar-Jaiswal *et al.* 2015b). Since MiMICs contain two *attP* sites and the donor vectors used for RMCE contain two *attB* sites, RMCE can occur in either orientation. Therefore, genes containing an intronic MiMIC in either orientation can be converted by RMCE to integrate useful cassettes.

The existing MiMIC collection of fly strains is a valuable tool for tagging genes at endogenous loci. The Gene Disruption Project generated ∼18,000 MiMIC insertions and deposited ∼7500 MiMIC insertions in the BDSC. Of these, 2854 are in coding introns of 1862 different genes (Nagarkar-Jaiswal *et al.* 2015b). All MiMIC lines are available from the BDSC and multiple tools to convert MiMIC insertions into tagged genes are available (see *RMCE resources for MiMIC or CRIMIC conversions*). In summary, the MiMIC collection is an off-the-shelf and readily available resource that can be used to tag 1862 genes with different tags. In addition, as shown in *CRISPR-based methods*, the MiMIC strategy can be expanded to many more genes using clustered regularly interspaced short palindromic repeats (CRISPR) technology.

### Reverse genetic tools

#### Homologous recombination:

Precise manipulation of target sequences through exploitation of homology-directed repair is now possible in many species (Peng *et al.* 2014). Homology-directed repair is one means by which cells may repair double-stranded breaks. In this pathway, exonucleases create protruding DNA overhangs in the region that contains the double-stranded break. These overhangs serve to invade homology-containing regions and replicate this region until a gap caused by the break is repaired (Jasin and Rothstein 2013). Often, the homologous chromosome serves as a template for the repair process, resulting in repair of the double-strand break. Alternatively, exogenous sequences with homology to the DNA near the break (donor construct) can be introduced into the cell to serve as a template for repair. In this case, the region contained between the homologous sequences is integrated in the locus where the double-strand break occurred. Depending on the donor construct, genes can be deleted, tagged, or replaced. Insertion of a dominant marker in the donor construct helps to identify the positive events.

Homologous recombination has been widely used to tag endogenous genes in bacteria and yeast. The technique was readily adapted in the 1980s in mouse biology through *in vitro* manipulation of embryonic stem cells (Capecchi 2005). In *Drosophila*, homologous recombination was adopted in 2000 by Golic and colleagues, who employed an elegant but complex strategy. A homology-containing donor construct is first inserted in the genome as a transgene. This construct is nested between two FRT sites and two 18-bp I-Sce restriction enzyme target sites. Upon expression of Flp and I-Sce, the donor construct is first circularized and excised from its initial insertion locus. Subsequently, I-Sce enzyme, expressed from a transgene, cuts and linearizes the donor construct, creating a recombinogenic substrate. The donor can then be integrated into the homologous locus (Rong and Golic 2000; Baena-Lopez *et al.* 2013; Chen *et al.* 2015). The complexity and time frame for generating homologous recombination alleles in *Drosophila* has limited their use, yet some laboratories became very well-versed in this technique. However, there are only 32 fluorescent protein-tagged genes available based on homologous recombination in the BDSC. The most systematic protein tagging effort, through homologous recombination, tagged 27 *Drosophila* Rab proteins with yellow fluorescent protein (YFP) (Dunst *et al.* 2015) or GAL4 (Chan *et al.* 2011). Although homologous recombination remains a viable and elegant tagging strategy, there are now faster and easier methods.

#### CRISPR-based methods:

Advances in CRISPR technology have made genome engineering much easier, faster, and cheaper. CRISPR-Cas9 nuclease can target sequences specified by 20 nucleotide (nt)-long guide RNAs (gRNAs). In flies, microinjection of Cas9 and a gRNA in developing embryos was shown to induce double-strand breaks and heritable mutations in a target locus at high frequency in germline cells. There are several strategies for Cas9 and gRNA delivery (Bassett and Liu 2014). We will only provide minimal information related to CRISPR here, as a separate chapter covers this topic in detail (Bier *et al.* 2017) (FlyBook chapter titled: Advances in Engineering the Fly Genome with the CRISPR-Cas system).

**Figure 7:**
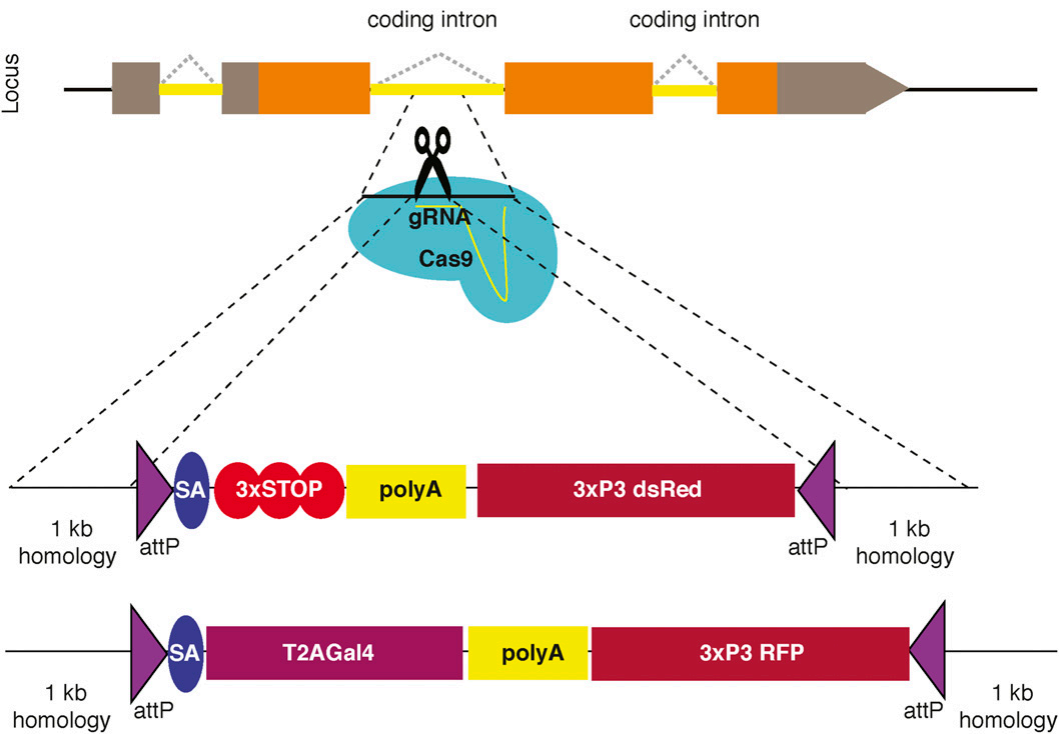
CRIMIC scheme. Coding intron sequence is cut by Cas9-gRNA complex. The sequences on either side of the cut site are used as 1-kb homology arms to integrate the SIC. Two possible SICs from published literature are included as examples. gRNA, guide RNA; RFP, red fluorescent protein; SIC, Swappable Insertion Cassette.

CRISPR-generated double-strand breaks can also be used for homology-directed repair. The size of the cassette to be integrated in the locus determines the nature of the donor DNA: single-stranded or double-stranded DNA. For shorter modifications (< 100 bp), such as insertion of a small epitope (*e.g.*, a HA or V5 tag) or an *attP* landing site, a single-stranded homology donor with ∼50 bp homology on either side of the cut site can be used. Since the inserted cassette is not large enough to contain a visible dominant marker, these insertions must be screened by PCR (Gokcezade *et al.* 2014; Gratz *et al.* 2014; Wissel *et al.* 2016). If a large construct needs to be integrated (> 100 bps) in the locus, a double-stranded plasmid donor with a 1-kb homology arm on either side of the cut DNA is necessary for integration. A dominant marker is usually present in these constructs to facilitate the detection of insertion events because of the low efficiency of integration (Gratz *et al.* 2013; Zhang *et al.* 2014; Diao *et al.* 2015) (Figure 7).

Recently, new methodologies were developed to introduce a MiMIC-like Swappable Insertion Cassette (SIC) in targeted introns by CRISPR (Zhang *et al.* 2014; Diao *et al.* 2015). The methods differ slightly between the two studies. Zhang *et al.* (2014) use two gRNAs to replace an exon with an artificial exon containing stop codons, polyA, and a dominant marker. Diao *et al.* (2015) use a single gRNA and integrate the artificial exon in the intron. Their artificial exon contains a T2A-GAL4. Both methods can be used to integrate tags, other genes, or constructs through RMCE (see *RMCE resources for MiMIC or CRIMIC conversions*), which is similar to MiMIC after the recovery of the original insertion. Recently, the *Drosophila* Genome Disruption Project stopped generating untargeted MiMIC insertions and switched to targeted insertions of MiMIC-like elements through CRISPR (CRIMIC). The aim of this project is to insert a CRIMIC cassette in 2500 genes that encode homologs of human genes and are not yet targeted by MiMIC. This endeavor will greatly facilitate tagging genes that may have diagnostic and therapeutic significance and accelerate the use of *Drosophila* in understanding human biology (Bellen and Yamamoto 2015).

### RMCE resources for MiMIC or CRIMIC conversions

**Figure 8:**
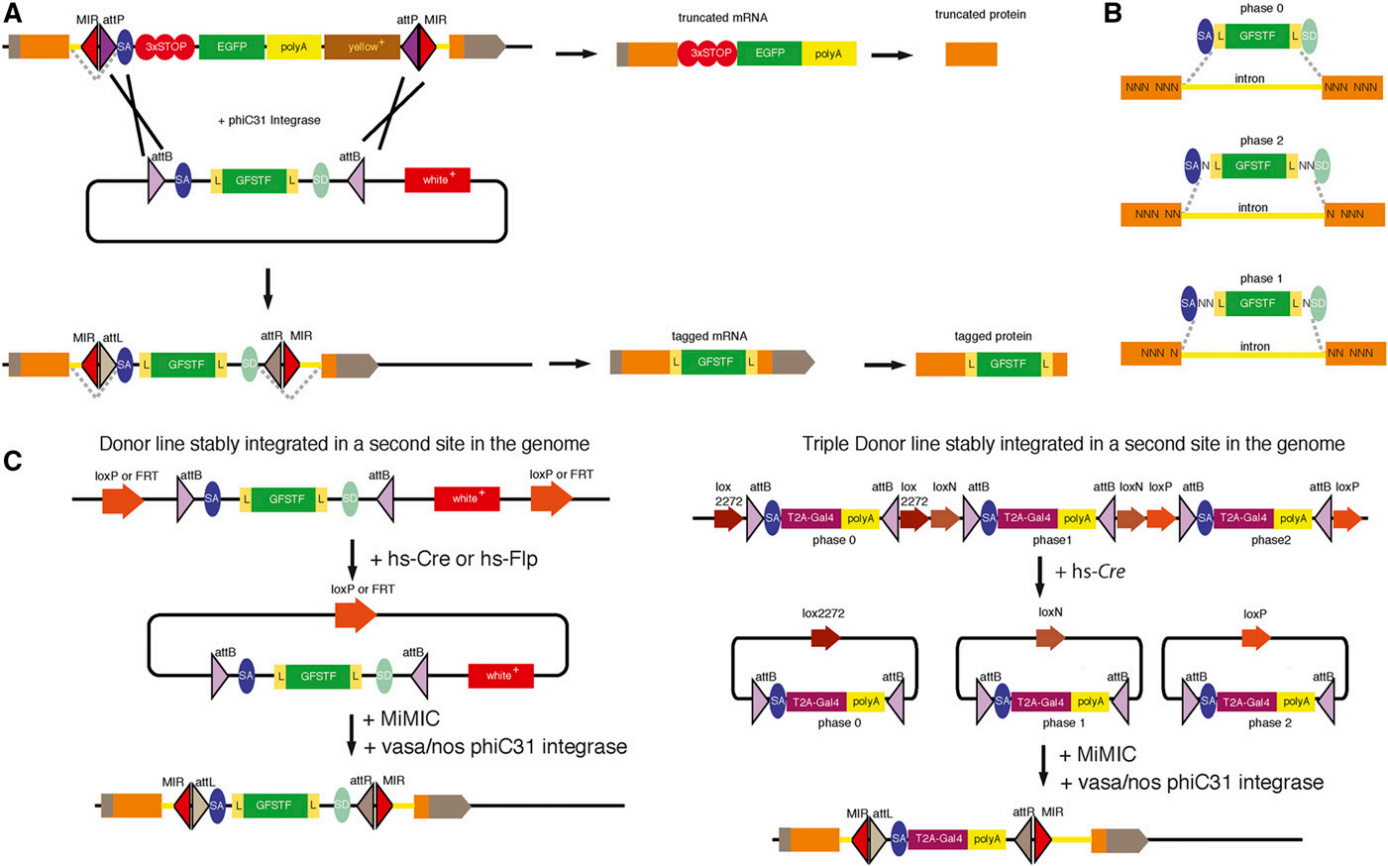
RMCE conversion scheme. (A) Conversion scheme of a MiMIC in the coding intron of a gene to tag the gene with GFP. Before conversion, MiMIC causes truncation of mRNA and protein. After conversion, mature mRNA will contain the artificial exon and the protein product will contain an internal GFP tag. (B) Explanation of possible open reading frames of a gene and how an RMCE cassette should be designed accordingly. N’s stand for nucleotides in the codons. (C) Different RMCE conversion strategies by crossing of fly strains. 3 × STOP, stop codons in all possible open reading frames; GSFTF, a multi-tag containing EGFP-FlAsH-StrepII-3XFlag and L flexible linkers; MiMIC, Minos-Mediated Integration Cassette; MIR, Minos Inverted Repeats; RMCE, Recombination-Mediated Cassette Exchange; SA, Splice Acceptor; yellow+, mini yellow dominant selection marker.

Site-specific recombinases and integrases are enzymes that catalyze recombination between their cognate DNA sequences. They serve as invaluable tools for precise genome editing in a variety of model organisms and contexts. Although several recombinase and integrase systems have been adapted for *Drosophila*, the most predominant ones remain the Flp/FRT, Cre/LoxP, and φC31 integrase systems (Golic 1991; Siegal and Hartl 1996; Groth *et al.* 2004). MiMICs contain an exchangeable cassette flanked by two *attP* sites that point toward each other (Figure 8A). Therefore, any DNA element nested between two similarly oriented *attB* sites can be used to replace the MiMIC cassette. This results in an exchange of the dominant marker *yellow+*, the stop codons, and polyA tail with the new cassette (Venken *et al.* 2011).

The RMCE process needs to take place in the germline of flies to be heritable. The necessary components are: (1) the φC31 integrase, (2) the RMCE donor construct, and (3) the MiMIC-carrying chromosome. Initial studies used a φC31 integrase mRNA microinjection for site-specific integration and RMCE (Groth *et al.* 2004; Bateman *et al.* 2006; Venken *et al.* 2006). However, currently, germline-specific expression of φC31 integrase is achieved by using *vasa* or *nanos* regulatory regions to express the recombinase in germ cells. *vasa/nos*-φC31 integrase can either be in a helper plasmid or in a transgene inserted in the fly genome (Bischof *et al.* 2007). An important consideration for inserting an artificial exon via RMCE is to select the correct open reading frame that corresponds to the open reading frame of the host gene. Since the exon–exon junction can occur in any of the three positions in a codon, the reading frame of the artificial exon is chosen according to the last codon of the exon it follows (Figure 8B). The RMCE donor cassette in a plasmid can be directly microinjected into embryos expressing the germline φC31 integrase and carrying the MiMIC insertion (Venken *et al.* 2011). Alternatively, RMCE donor cassettes can be mobilized *in vivo* from transgenes. The latter eliminates the need for microinjection but does not necessarily improve the efficiency of RMCE. *In vivo* mobilization of the RMCE cassette is accomplished by generating transgenes in which the RMCE cassette is flanked by directional FRT or LoxP sites (Diao *et al.* 2015; Nagarkar-Jaiswal *et al.* 2015a). The expression of the corresponding site-specific recombinase causes circularization and excision of the RMCE cassette that serves as donor DNA. MiMIC elements can be crossed into a genetic background where the RMCE cassette can be mobilized and integrated in the MiMIC-bearing chromosome in germ cells (Figure 8C).

The choice of RMCE cassette depends on the desired application. If the aim is to generate a functional tagged protein, the RMCE cassette should not be mutagenic. Many RMCE cassettes that replace the MiMIC cassette with an SA-flexible linker (L)-Fluorescent Protein (FP)-flexible linker-SD) are available (Venken *et al.* 2011; Nagarkar-Jaiswal *et al.* 2015a,b). These constructs do not contain stop codons or a polyA tail, and are not intrinsically mutagenic. However, introducing a protein such as EGFP internally in another protein can be mutagenic as it potentially disrupts functional domains or blocks protein interaction surfaces. However, this potential negative effect can be attenuated by the use of flexible linkers before and after the fluorescent tags, allowing for structural flexibility. Moreover, bioinformatics analysis of multiple metazoan genomes, including *Drosophila*, showed that the DNA elements that encode functional protein domains are predominantly not divided by introns (Liu and Grigoriev 2004). Consistent with this observation, Nagarkar-Jaiswal *et al.* (2015) analyzed the effect of a SA-L-EGFP-FlAsH-StrepII-3 × Flag-L-SD cassette on functionality of the host gene when inserted in coding introns (Nagarkar-Jaiswal *et al.* 2015b). The internal EGFP insertion did not obviously affect protein function in 75% of 114 essential genes tagged. Further analysis showed that functional integral tags were biased to not bisect functional protein domains. Preselection of coding introns that do not bisect annotated functional domains for CRIMIC insertions can further increase the chance of obtaining functional tags. In summary, integral tagging of genes via MiMIC and RMCE often does not impair protein functionality, provided that the coding intron does not separate important functional protein domains.

Sometimes, it is preferable to maintain the mutagenicity of MiMIC while inserting new transgenes through RMCE. RMCE cassettes that contain SA-T2A-effector protein-polyA are designed for this purpose. T2A is an 18-aa long peptide of viral origin that, during translation, breaks the continuity of peptide bonds (Donnelly *et al.* 2001; Diao and White 2012). This results in the release of the nascent, truncated protein at the T2A site but allows the translation of sequences following T2A and the production of a new polypeptide. Thus, the SA-T2A-effector protein-polyA cassette causes the truncation of the host gene product similar to the MiMIC allele but, in addition, expresses an effector protein (*e.g.*, GAL4, LexA, Flp, or GAL80) from the same mRNA (Diao *et al.* 2015; Gnerer *et al.* 2015). The SA-T2A-GAL4-polyA cassette is the most widely used, since it offers a GAL4 driver that is expressed with the same spatial and temporal properties as the gene that it mutates. The GAL4 can then be used to drive the UAS-gene of interest (*e.g.*, GFP); this greatly facilitates the detection of gene expression patterns and permits the replacement of the mutated gene with a variant of the gene, for example an ortholog from another species (*e.g.*, human ortholog) (Bellen and Yamamoto 2015). Diao *et al.* (2015) used the ease of T2A-GAL4 detection to devise an elegant strategy for facilitating RMCE crosses. Their donor DNA transgene contains three copies of T2A-GAL4, each copy corresponding to a different reading frame. These T2A-GAL4 cassettes are flanked by compatible Lox sites that differ for each construct, so that the expression of Cre simultaneously mobilizes each of the cassettes. Only the RMCE with a T2A-GAL4 cassette of the correct reading frame and in the correct orientation results in functional GAL4. Flies where an RMCE event can occur in the germline are crossed to UAS-GFP, and the resulting larvae are easily screened for GFP expression, which indicates correct RMCE events. With this strategy, it is possible to mobilize the donor construct from the same transgenic fly for any gene (Figure 8C).

Recently, two techniques that use MiMICs were developed to generate conditional mutant alleles. They both use the FLP/FRT system to change the direction of a cassette from a nonmutagenic to a mutagenic orientation. Flp-Stop uses a UAS-tomato reporter gene that can only be activated when the construct is in the mutagenic conformation (Fisher *et al.* 2017). Flip-Flop uses a compound cassette that tags the gene with GFP when it is in a nonmutagenic conformation, the protein trap orientation. Upon FLP expression, the cassette inverts to the gene trap orientation introducing a T2A-mCherry with a polyA tail, thereby creating a severe loss-of-function mutation (Nagarkar-Jaiswal *et al.* 2017).

In summary, RMCE renders MiMIC and MiMIC-like elements very versatile. By converting the same initial insertion into different tags (*i.e.*, fluorescent protein tags, biochemical epitopes, T2A-GAL4 drivers, or T2A-GAL80 repressors), a gene can be thoroughly characterized and annotated.

## Applications for Epitope Tags

In this section, we focus on techniques that utilize tags to visualize, purify, or modify the tagged proteins. Prior to detailing the applications, we discuss important controls to obtain meaningful data using epitope tags. There are two main caveats to the use of epitope tags: the tagged gene may be nonfunctional and the expression levels of the tag may be nonphysiological. First, the functionality of a tagged gene should be tested. A rescue of the mutant phenotype associated with loss-of-function mutations of the gene of interest with the tagged construct ensures that important functional interactions of the tagged protein are not impaired. If there are no known phenotypes associated with the loss-of-function of the gene of interest it is difficult to address the functionality of the tagged transgene. Second, the expression level should not deviate significantly from the endogenous expression level. When a tagged gene is expressed at nonphysiological levels, the encoded protein may interact with partners that it does not normally interact with, accumulate at subcellular locations where it does not normally localize, or both. This may cause false positive interactors and artifactual phenotypes. Hence, controls need to be included in the experimental design prior to using a tagged gene for the following applications.

### Protein visualization *in vivo*

#### Immunofluorescent detection:

To assess the function of a gene or the protein that it encodes, it is important to know which cells express the gene/protein at which stage and where the protein localizes subcellularly. The standard approach to answer these questions is immunohistochemical analysis. However, few reliable primary antibodies against *Drosophila* proteins are available. The availability of numerous tagged transgenic clones in the TRG library allow us now to systematically determine protein expression and localization during fly development and in adult flies. This has so far been most systematically done during oogenesis and in the adult thorax using fixed samples. In particular, the large cells of the egg chamber and adult flight muscles reveal a multitude of subcellular localization, suggestive of possible functions (Spletter *et al.* 2015; Sarov *et al.* 2016) (Figure 5C). The ability to use the same staining protocol with the same antibody allows for a relatively high sample throughput without compromising robust detection. This should inspire a variety of other systematic analyses of other tissues or developmental stages in the near future.

#### Live imaging and optic manipulations:

The development of every organism and the physiology of every cell relies on dynamic processes. These processes often require protein synthesis, transport, anchoring, and turnover, features that often escape fixed immunohistochemical analysis. The discovery of GFP and its derivatives were revolutionary, as it enabled protein visualization in real time through the use of special sensitive fluorescent microscopes. Quick, ingenious ways of using fluorescence to detect protein colocalization, binding, half-life, mobility, and diffusion rates were developed. Thus, tagging a gene with a fluorescent tag provides very valuable information about diverse aspects of protein dynamics. For detailed information related to this topic we refer to the FlyBook chapter Protein and RNA imaging technologies.

The TRG library lines can be used for live imaging. The folding of sGFP, used in this library, is significantly faster than the regular GFP (Pédelacq *et al.* 2006), making it possible to do live imaging in embryos. Indeed, the dynamic expression of some transcription factors during neuroblast development can be imaged in living embryos (Sarov *et al.* 2016), and we anticipate that it may be possible to track many cells throughout embryogenesis using these imaging tools. Similarly, live imaging during pupal development has been documented with TRG clone-bearing flies, again revealing dynamic patterns for many proteins (Sarov *et al.* 2016). As pupae do not move, they are particularly well-suited for live imaging and the routine application of multi-photon imaging enabling enhanced penetration depth will further promote the study of dynamic morphogenetic processes during pupal development (Weitkunat *et al.* 2014, 2017).

There is not a single tag that optimally fits all live imaging applications. However, fluorescent tags encoded by GFP or Cherry are continuously being adapted and improved (Cranfill *et al.* 2016). The sGFP folds very quickly, but still retains a small tendency to dimerize, a potential problem of a number of fluorescent proteins (Costantini *et al.* 2015). Hence, functionality of the tagged proteins needs to be tested. MiMIC insertions enable the efficient exchange of fluorescent tags *in vivo*, making them useful to test newly developed fluorescent protein variants (Nagarkar-Jaiswal *et al.* 2015a). In addition, improvements in microscopy technology, including light sheet microscopy, have enabled imaging of all cells of developing embryos (Huisken *et al.* 2004). Moreover, two-photon microscopy allows imaging in thick living samples, thereby opening up new avenues to apply live imaging to the fly (see FlyBook chapter Protein and RNA imaging technologies).

Technology development also enables optical manipulations. Laser-induced microlesions *in vivo* are the method of choice to quantitatively determine mechanical damage in living tissue (Colombelli *et al.* 2005; Solon *et al.* 2009; Weitkunat *et al.* 2014). This requires the expression of fluorescent proteins to mark the tissues of interest. A direct measure of protein dynamics is often achieved by fluorescence recovery after photobleaching, which can for example measure receptor turnover rates *in vivo* (Pines *et al.* 2012). In addition, direct inactivation of GFP-tagged proteins has been reported by a method dubbed CALI (chromophore-assisted laser inactivation). During CALI, a strong laser intensity is applied to produce radicals in close proximity to the tagged protein, which often cross-links, and hence acutely inactivates the tagged protein. This allows assessment of the function of the tagged protein in real time (Monier *et al.* 2010). An additional variant of fluorescence-induced protein inactivation uses a tetra-cysteine tag in addition to the GFP. This tag binds to a cell-permeable fluorescein variant (FlAsH), which upon laser illumination produces a large amount of radicals, acutely inactivating the tagged protein (Marek and Davis 2002; Venken *et al.* 2008). As both inactivation methods use a rather high laser dose, adequate controls are essential. In summary, this brief overview illustrates how live imaging expands the potential of tagged proteins that are typically under the control of regulatory elements of the corresponding gene.

### Biochemical uses of tags

An important aspect of the annotation of a gene is the function of the gene product. Detection of expression domains and the subcellular localization of proteins does not provide direct information about the function of the gene. In depth functional analysis typically relies, in addition to phenotypic analysis of mutants, on biochemical methods for purification of the protein of interest and its interaction partners. Immunoprecipitation is one of the most commonly used methods to purify proteins and their interactors (*i.e.*, nucleic acids, proteins, or other macromolecules). Immunoprecipitation methods typically use an antibody that binds to the protein of interest and is coupled to a solid substrate (typically beads or columns) to enable physical separation of an antibody–protein complex from the sample solution (*e.g.*, cell lysate). The success of an immunoprecipitation experiment depends largely on the quality of the used antibody.

Antibodies against the endogenous proteins are typically raised through immunizing an animal and purifying the antibody from the serum of the immunized animal. The first limitation of immunization is that some of the epitopes used for generation of the antibody are poorly immunogenic and the produced antibodies have low affinity and specificity for the protein. For example, empirical findings after thousands of chromatin immunoprecipitation (ChIP) experiments indicate that only 10% of antibodies raised against endogenous proteins are usable for immunoprecipitation of nucleic acid–protein complexes (Savic *et al.* 2015). Second, most antibodies are polyclonal and hence exhaustible. Third, the conditions for each antibody must be optimized. Finally, every precipitation experiment needs controls to ensure specificity. The best control is to use lysates devoid of the protein of interest, such as lysates from mutant cells or animals, but mutant samples can be difficult to obtain due to lethality. In summary, antibodies provided us with great tools but the fact that they are a scarce resource for immunoprecipitation is a major drawback.

Specific epitopes like GFP allow the use of well-characterized and commercially available antibodies against the epitope tag. Even when different proteins are detected or manipulated, the use of the same epitope tag standardizes experimental methods. Moreover, the wild-type cells or animals that do not contain the tag can serve as controls. These advantages make the use of epitope tags for immunoprecipitation experiments the method of choice for protein–protein or protein–nucleic acid interactions.

The detection of bound proteins, RNA, or DNA depends on the nature of the interactor. For protein–nucleic acid interactions, the bound nucleic acid needs to be sequenced. If the pull-down experiment aims to quantify a known interaction, qPCR with interactor-specific primers can be used. If no prior information is present for the interaction, then nucleic acid sequencing (ChIP-seq) or microarray hybridization (ChIP-on-chip) are the methods of choice (Furey 2012). The modEncode project systematically used multiple GFP-tagged transcription factors in P[acman] constructs for pull-down experiments to catalog the transcription factor-binding sites (The modENCODE Consortium *et al.* 2010; Negre *et al.* 2011).

For protein–protein interactions, western blots allow detection and quantification of known interactions. Epitope tags are especially useful to test whether a protein homopolymerizes by expressing two constructs that tag the gene with two different epitope tags. One of the tags can be used for immunoprecipitation and the other one for detection to test homopolymerization. When there is no prior knowledge about the interactors, mass spectroscopy is a powerful technique to identify and quantify interactors, as well as determine complexes associated with the protein of interest (Neumüller *et al.* 2012; Zhang *et al.* 2013; David-Morrison *et al.* 2016; Yoon *et al.* 2017).

### Tag-mediated gene inactivation schemes

**Figure 9:**
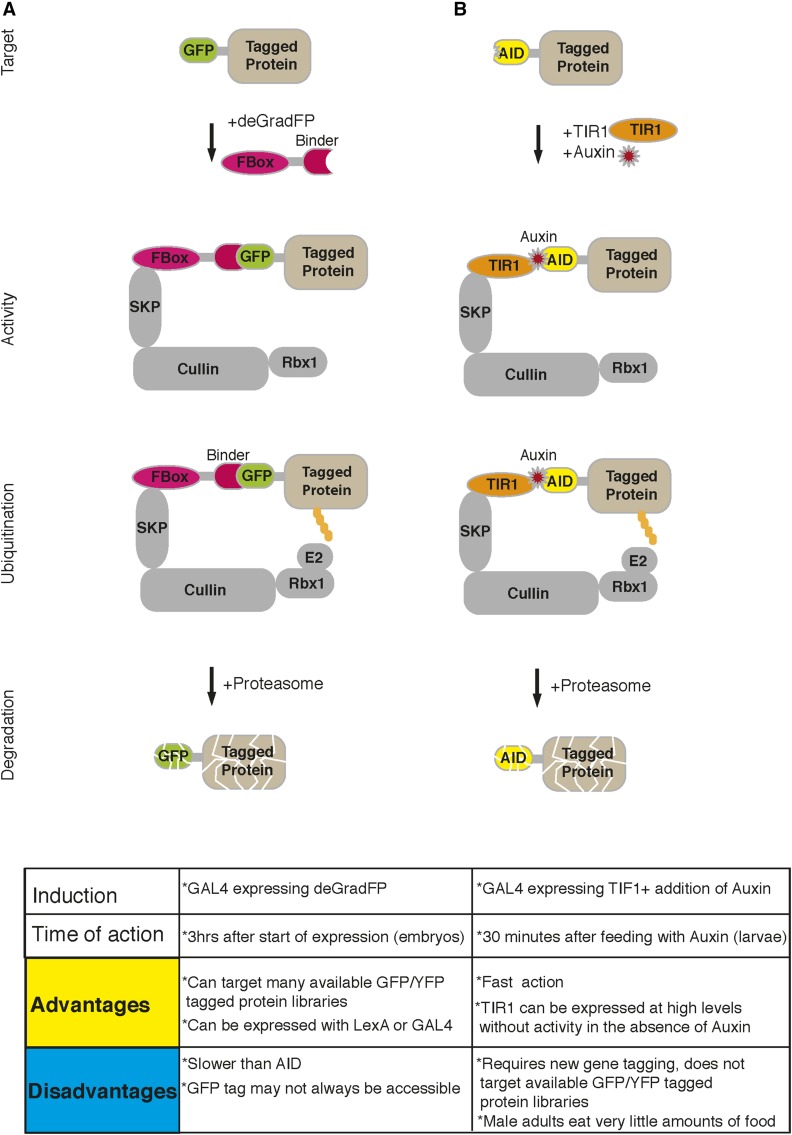
Schematic mode of action for protein removal tools. (A) deGradFP construct binds to GFP-tagged proteins constitutively through peptide binder and recruits the ubiquitination machinery to the target. Ubiquitinated protein is often degraded by the proteasome. (B) Auxin-induced degradation (AID) works by the TIR1 binding to AID in an Auxin-dependent manner. In the absence of Auxin, TIR does not bind to AID-tagged protein. Auxin causes rapid TIP1 binding to AID-tagged protein, which recruits the ubiquitination machinery. Ubiquitinated protein is often degraded by the proteasome. YFP, yellow fluorescent protein.

Once a gene is tagged, an RNA interference construct or an antibody against the tag can be developed to impair the function of the gene product. The most commonly targeted tag is GFP. There are major advantages to targeting GFP for gene inactivation: (1) knockdown can be visualized in real time; (2) the phenotype can be correlated with the level of knockdown; (3) tissues that express GFP, in which the gene is not knocked down, serve as controls; (4) animals that do not contain the GFP tag but express the inactivation tool can serve as specificity control for the manipulation; and (5) a single well-characterized toolkit can be used to target all the tagged genes/proteins. There are two main methods to knockdown GFP-tagged genes/proteins: (1) iGFPi, which uses a well-characterized, potent short hairpin RNA with minimal off-target effects to degrade GFP-containing transcripts (Neumüller *et al.* 2012; Wissel *et al.* 2016); and (2) deGradFP, a protein that binds GFP and leads to degradation or inactivation of the target protein. The moiety of deGradFP that recognizes GFP encodes a 14 kDa nano-antibody, also called a protein binder. Protein binders are typically short peptides that bind to a target protein with high affinity. Different strategies exist to generate peptide binders and a discussion of the different binders is beyond the scope of this chapter (Bieli *et al.* 2016). The key issue is that peptide binders are short polypeptides that can be produced intracellularly by a transgene. For example, in the deGradFP, the peptide binder against GFP is fused to the F-Box domain of E3 ubiquitin-ligase *slmb* (Caussinus *et al.* 2012). Normally, the F-Box proteins confer substrate specificity to the ubiquitination machinery. They bind to substrates with special domains to mark them for degradation. In the deGradFP constructs, the substrate recognition domain of *slmb* is replaced by a peptide binder that recognizes GFP. In the presence of a GFP-tagged substrate, deGradFP binds and recruits the ubiquitination machinery to ubiquitinate the tagged protein so that it can be degraded by the proteasome (Caussinus *et al.* 2012) (Figure 9A). Although the deGradFP acts at the protein level and the iGFPi acts at the RNA or translational level, both should be tried in parallel if possible. For example, the *dunce* gene was tagged with GFP and the GFP was expressed in the mushroom bodies as anticipated. This allowed the conditional removal of the tagged protein using a mushroom body GAL4 driver for UAS-deGradFP expression in adult flies. The flies become dunce when they lose the tagged Dunce protein in MB. Interestingly, by playing with temperature conditions that regulate GAL4-dependent deGradFP expression, smart flies were made dunce and smart again (Nagarkar-Jaiswal *et al.* 2015b).

A different approach to conditionally deplete proteins of interest is to use more specialized tags to destabilize tagged proteins. These tags are commonly referred to as degrons and lead to proteasome-mediated degradation when they are fused to proteins. There are three main strategies to make degrons conditional: (1) use a small molecule to mask the degron (Banaszynski *et al.* 2006; Iwamoto *et al.* 2010); (2) mask the degron with a peptide moiety that can be cleaved off by a site-specific protease (TIPI; Taxis *et al.* 2009); and (3) use a degron that depends on a small molecule for activity (Chung *et al.* 2015; Trost *et al.* 2016; Bence *et al.* 2017) (Figure 9B). Some of these methods may act faster than deGradFP and iGFPi but cannot be used in combination with available GFP-tagged genetic libraries and require the generation of new, specialized transgenic constructs.

An alternative technique to impair protein function is to sequester them. This is especially useful to study morphogens, signaling molecules whose tissue distribution directly affects patterning of the tissue. Morphotrap is a peptide binder fused to a transmembrane protein such as CD8. In this construct, the peptide binder is exposed to the extracellular space to trap extracellular GFP-tagged proteins and alter their tissue distribution either at the source of the morphogen or at the target tissue (Harmansa *et al.* 2015). A similar technique to sequester cytoplasmic proteins in apical or basal domains of polarized cells, GrabFP, was recently developed (Harmansa *et al.* 2017). In summary, a tagged gene facilitates the use of ever increasing precision tools to degrade, mis-localize, or sequester gene products.

## Conclusions and Outlook

Functional tags are extremely valuable tools. Currently, the use of the methods summarized here allows the determination of expression and the functional manipulation of ∼2500 individual genes based on available fly strains. This number is likely to significantly increase in the near future as both CRIMIC, FlyFos, and FlyORF stocks are being created.
